# State-Specific Hepatitis C Virus Clearance Cascades — United States, 2013–2022

**DOI:** 10.15585/mmwr.mm7321a4

**Published:** 2024-05-30

**Authors:** Clarisse A. Tsang, Julius Tonzel, Hasan Symum, Harvey W. Kaufman, William A. Meyer, Ademola Osinubi, William W. Thompson, Carolyn Wester

**Affiliations:** ^1^Division of Viral Hepatitis, National Center for HIV, Viral Hepatitis, STD, and TB Prevention, CDC; ^2^Department of Epidemiology, School of Public Health, Louisiana State University Health Sciences Center, New Orleans, Louisiana; ^3^Quest Diagnostics, Secaucus, New Jersey.

**Summary**

**What is already known about this topic?**

Hepatitis C is a deadly, yet curable, disease. National goals for 2030 call for at least 80% of persons with hepatitis C to achieve viral clearance through well-tolerated, highly effective treatment.

**What is added by this report?**

Analysis of 2013–2022 data from a large national, commercial laboratory found that hepatitis C viral clearance proportions among persons with hepatitis C varied by state from 10% to 51% and fell below established hepatitis C viral clearance goals in all jurisdictions.

**What are the implications for public health practice?**

The assessment of variations in hepatitis C testing and treatment can help identify gaps, prioritize activities to improve linkage to treatment and prevention services, and allocate resources for state hepatitis C elimination programs.

## Abstract

Hepatitis C is a deadly, yet curable, disease. National hepatitis C elimination goals for 2030 call for at least 80% of persons with hepatitis C to achieve viral clearance. A well-tolerated treatment results in sustained viral clearance in ≥95% of cases. Hepatitis C virus (HCV) clearance cascades characterize a sequence of steps that follow the progression from testing to sustained viral clearance. Monitoring HCV clearance cascades is important for tracking progress toward elimination goals and identifying gaps in diagnosis, treatment, and prevention. State-specific HCV clearance cascades based on laboratory results were developed using longitudinal data from a large national, commercial laboratory during January 1, 2013–December 31, 2022. State-level estimates of viral testing among persons with evidence of past or current HCV infection ranged from 51% (Hawaii) to 99% (South Dakota), and hepatitis C viral clearance among persons with diagnosed HCV infection ranged from 10% (West Virginia) to 51% (Connecticut). These are the first state-level estimates using CDC guidance and data from a large commercial laboratory with national coverage to generate HCV clearance cascades. These estimates reveal substantial gaps in hepatitis C diagnosis, treatment, and prevention and can help guide prioritization of activities and resources to achieve hepatitis C elimination goals.

## Introduction

During January 2017–March 2020, approximately 2 million adults in the United States were estimated to be infected with hepatitis C virus (HCV) (*1*), and new infections approximately doubled from 2013–2022, primarily in association with injection drug use (*2*). Untreated, HCV infection can lead to advanced liver disease, liver cancer, and death; hepatitis C screening is recommended for all adults (*3*). An 8–12-week course of well-tolerated, oral treatment with direct-acting antiviral (DAA) agents is recommended for nearly all persons with HCV infection (*4*); treatment results in sustained viral clearance in ≥95% of cases (*5*), making elimination of hepatitis C as a public health threat feasible. The U.S. Department of Health and Human Services (HHS) 2021–2025 Viral Hepatitis National Strategic Plan (*6*) provides a framework for hepatitis C elimination in the United States and calls for increasing the percentage of persons who have cleared HCV infection to at least 58% by 2025 and 80% by 2030.

Substantial variation exists among states with respect to hepatitis C disease incidence and public policies affecting access to hepatitis C treatment and prevention services for persons with or at risk for acquiring hepatitis C. The HCV clearance cascade process quantifies the proportions of persons with HCV at the following five steps: 1) those who were ever infected with HCV, 2) those who received complete (e.g., HCV RNA) testing, 3) those who were identified as having an initial infection, 4) those who subsequently demonstrated viral clearance either spontaneously or in response to treatment, and 5) among those who initially cleared the virus, subsequently had evidence of recurrent viremia because of either persistent infection (e.g., unsustained viral clearance because of treatment failure) or reinfection because of ongoing risk for acquiring hepatitis C. Each state should characterize its own HCV clearance cascade to monitor progress toward state-specific hepatitis C elimination goals and prioritize allocation of public health resources.

In 2021, CDC published guidance for developing a simplified HCV clearance cascade based on HCV laboratory test results, such as those contained in public health surveillance systems (*7*). However, many state public health surveillance systems do not include comprehensive HCV test results or lack the ability to receive, deduplicate, and track person-level longitudinal laboratory test results, precluding the development of state-level HCV clearance cascades. Longitudinal commercial laboratory results have been used to develop a national HCV clearance cascade (*8*). The primary goal of this study was to develop state-specific HCV clearance cascade estimates to assist states in identifying opportunities to diagnose, treat, and prevent HCV infections in their jurisdiction.

## Methods

### Data Source and Definitions

Ten years of data (January 1, 2013–December 31, 2022) were analyzed for patients in all 50 states and the District of Columbia (DC) who received HCV testing by Quest Diagnostics (https://www.questdiagnostics.com). Quest Diagnostics programming was applied to deidentify and deduplicate data. Using client or provider zip code data from the laboratory requisition and a hierarchical algorithm, researchers assigned persons to a state in the following order: 1) client data from the first HCV test result of the cascade (92%), 2) ordering provider's data from the first HCV test result of the cascade (8%), and 3) client data from any subsequent HCV test result in the cascade (<1%). Test results included HCV antibody (anti-HCV), HCV RNA (quantitative or qualitative), and HCV genotype. Using previously published CDC guidance (*7*), state-specific HCV clearance cascades characterized persons according to five criteria: 1) ever infected (having received any positive HCV test result [reactive anti-HCV, detectable HCV RNA or HCV genotype] during January 1, 2013–December 31, 2021 [index period]); 2) received viral testing (having had an HCV RNA test performed during January 1, 2013–December 31, 2022, among persons categorized as ever infected [follow-up period]); 3) diagnosis of initial infection (having a detectable HCV RNA test result during the follow-up period for any person with viral testing); 4) cured or cleared (having received a subsequent undetectable HCV RNA test result during the follow-up period among any person with an initial infection); and 5) persistent infection or reinfection (having received a subsequent detectable HCV RNA test result during the follow-up period in any person categorized as cured or cleared).

### Data Analysis

Frequencies at each step of the clearance cascade were calculated and stratified by state. Conditional proportions for each step of the clearance cascade were calculated using methods similar to those in the CDC Laboratory-based Hepatitis C Virus Clearance Cascade: Program Guidance for Local and State Health Departments document (*7*). Jurisdictional proportions were suppressed using National Center for Health Statistics reporting guidelines, and Clopper Pearson 95% CI estimates were also calculated. For the estimated state-level proportions, data were assumed to be missing at random.* Analyses were performed using RStudio (version 4.2.2; RStudio). This activity was reviewed by CDC, deemed research not involving human subjects, and was conducted consistent with applicable federal law and CDC policy.^†^

## Results

### State-Specific Clearance Cascade Proportions

**FIGURE 1:**
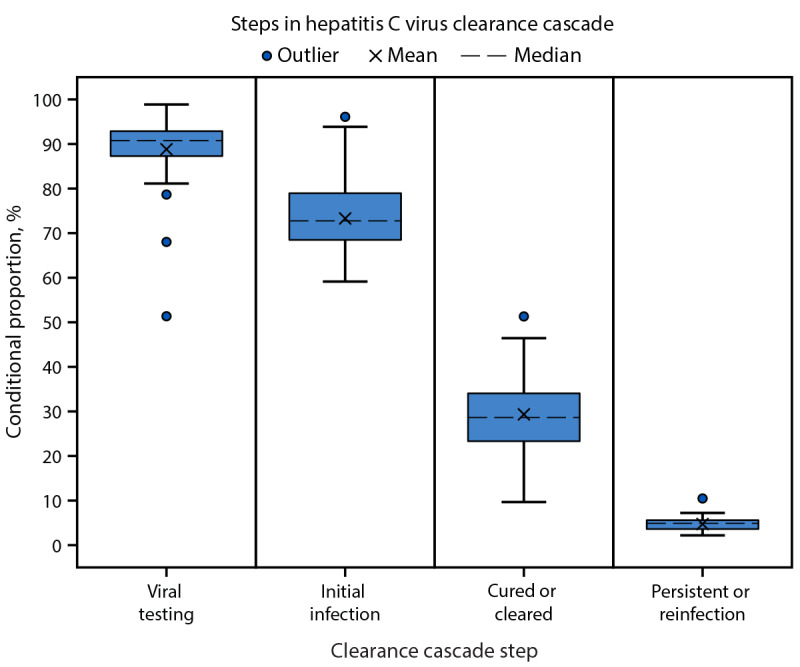
State-level hepatitis C virus clearance cascade estimates* — United States, 2013–2022^†^^,^^§^^,^^¶^ **Abbreviation:** HCV = hepatitis C virus. * Conditional proportion based on immediately preceding cascade step. Quartiles are calculated using the inclusive median. ^†^ Includes all persons ever infected during the baseline period January 1, 2013–December 31, 2021. ^§^ The viral testing, initial infection, cured or cleared, and persistent infection or reinfection categories were assessed during the follow-up period of January 1, 2013–December 31, 2022. ^¶^ Using CDC guidance (https://pubmed.ncbi.nlm.nih.gov/32119076/), state-specific HCV clearance cascades were generated using the following definitions: 1) ever infected, defined as a person receiving any positive HCV test result (reactive anti-HCV, detectable HCV RNA, or HCV genotype) during January 1, 2013–December 31, 2021 (index period); 2) viral testing, defined as a person receiving HCV RNA testing during January 1, 2023–December 31, 2022 (the follow-up period) for a person categorized as ever infected; 3) initial infection, defined as having received a detectable HCV RNA result during the follow-up period for any person who received viral testing; 4) cured or cleared, defined as having received a subsequent undetectable HCV RNA result during the follow-up period for any person with initial infection; and 5) persistent infection or reinfection, defined as receiving a subsequent detectable HCV RNA result during the follow-up period by person categorized as cured or cleared.

Among the sample of 1,631,609 unique patients identified as having ever been infected, 1,627,170 (99.7%) had available state information from the laboratory requisition forms (Table). Across all states, the median proportions of viral testing, initial infection, cured or cleared, and persistent infection or reinfection were 91%, 73%, 29% and 5%, respectively (Figure 1).

**TABLE:** Hepatitis C virus clearance cascade, by jurisdiction*^,†,§,¶^ — United States, 2013–2022

Jurisdiction	No. ever infected^†^	Viral testing^§^		Initial infection^§^		Cured or cleared^§^		Persistent infection or reinfection^§^	
		No.	% (95% CI)**	No.	% (95% CI)**	No.	% (95% CI)**	No.	% (95% CI)**
**Total**	**1,627,170**	**1,455,895**	**89.5 (89.4–89.5)**	**1,015,147**	**69.7 (69.6–69.8)**	**350,296**	**34.5 (34.4–34.6)**	**23,685**	**6.8 (6.7–6.8)**
Alabama	19,538	18,023	92.2 (91.9–92.6)	13,176	73.1 (72.5–73.8)	3,072	23.3 (22.6–24.0)	127	4.1 (3.5–4.9)
Alaska	6,752	5,760	85.3 (84.4–86.1)	4,560	79.2 (78.1–80.2)	1,534	33.6 (32.3–35.0)	98	6.4 (5.2–7.7)
Arizona	67,995	59,416	87.4 (87.1–87.6)	42,584	71.7 (71.3–72.0)	13,966	32.8 (32.4–33.2)	939	6.7 (6.3–7.2)
Arkansas	14,586	13,610	93.3 (92.9–93.7)	10,864	79.8 (79.1–80.5)	2,641	24.3 (23.5–25.1)	85	3.2 (2.6–4.0)
California	338,715	303,634	89.6 (89.5–89.7)	214,377	70.6 (70.4–70.8)	83,337	38.9 (38.7–39.1)	8,724	10.5 (10.3–10.7)
Colorado	18,635	17,425	93.5 (93.1–93.9)	11,675	67.0 (66.3–67.7)	2,984	25.6 (24.8–26.4)	76	2.5 (2,3–2.0)
Connecticut	36,389	34,485	94.8 (94.5–95.0)	20,774	60.2 (59.7–60.8)	10,660	51.3 (50.6–52.0)	619	5.8 (5.4–6.3)
DC	4,351	2,960	68.0 (66.6–69.4)	2,056	69.5 (67.8–71.1)	257	12.5 (11.1–14.0)	15	5.8 (3.3–9.4)
Delaware	4,052	3,820	94.3 (93.5–95.0)	3,050	79.8 (78.5–81.1)	708	23.2 (21.7–24.8)	28	4.0 (2.6–5.7)
Florida	191,214	175,887	92.0 (91.9–92.1)	114,913	65.3 (65.1–65.6)	49,057	42.7 (42.4–43.0)	3,353	6.8 (6.6–7.1)
Georgia	41,073	38,128	92.8 (92.6–93.1)	25,882	67.9 (67.4–68.4)	7,886	30.5 (29.9–31.0)	470	6.0 (5.4–6.5)
Hawaii	222	114	51.4 (44.6–58.1)	107	93.9 (87.8–97.5)	—^††^	—	—	—
Idaho	2,684	2,566	95.6 (94.8–96.3)	2,069	80.6 (79.0–82.1)	784	37.9 (35.8–40.0)	24	3.1 (2.0–4.5)
Illinois	33,881	30,389	89.7 (89.4–90.0)	20,677	68.0 (67.5–68.6)	6,253	30.2 (29.6–30.9)	315	5.0 (4.5–5.6)
Indiana	16,696	15,469	92.7 (92.2–93)	12,614	81.5 (80.9–82.2)	3,142	24.9 (24.2–25.7)	122	3.9 (3.2–4.6)
Iowa	3,483	3,138	90.1 (89.1–91.1)	2,456	78.3 (76.8–79.7)	870	35.4 (33.5–37.4)	37	4.3 (3.0–5.8)
Kansas	9,276	8,793	94.8 (94.3–95.2)	6,449	73.3 (72.4–74.3)	2,981	46.2 (45.0–47.5)	146	4.9 (4.2–5.7)
Kentucky	42,249	38,106	90.2 (89.9–90.5)	28,359	74.4 (74.0–74.9)	6,492	22.9 (22.4–23.4)	469	7.2 (6.6–7.9)
Louisiana	22,773	20,667	90.8 (90.4–91.1)	14,815	71.7 (71.1–72.3)	4,385	29.6 (28.9–30.3)	172	3.9 (3.4–4.5)
Maine	2,660	2,433	91.5 (90.3–92.5)	1,916	78.8 (77.1–80.4)	624	32.6 (30.5–34.7)	15	2.4 (1.4–3.9)
Maryland	33,507	28,261	84.3 (84.0–84.7)	18,810	66.6 (66.0–67.1)	5,415	28.8 (28.1–29.4)	287	5.3 (4.7–5.9)
Massachusetts	70,380	62,536	88.9 (88.6–89.1)	45,231	72.3 (72.0–72.7)	16,924	37.4 (37.0–37.9)	1,161	6.9 (6.5–7.3)
Michigan	27,506	24,549	89.2 (88.9–89.6)	16,987	69.2 (68.6–69.8)	3,652	21.5 (20.9–22.1)	197	5.4 (4.7–6.2)
Minnesota	4,609	4,084	88.6 (87.7–89.5)	2,435	59.6 (58.1–61.1)	511	21.0 (19.4–22.7)	19	3.7 (2.3–5.7)
Mississippi	6,135	5,525	90.1 (89.3–90.8)	4,615	83.5 (82.5–84.5)	1,276	27.6 (26.4–29.0)	45	3.5 (2.6–4.7)
Missouri	37,813	34,764	91.9 (91.7–92.2)	26,122	75.1 (74.7–75.6)	9,897	37.9 (37.3–38.5)	460	4.6 (4.2–5.1)
Montana	2,456	2,196	89.4 (88.1–90.6)	1,599	72.8 (70.9–74.7)	359	22.5 (20.4–24.6)	9	2.5 (1.2–4.7)
Nebraska	2,474	2,025	81.9 (80.3–83.4)	1,235	61.0 (58.8–63.1)	242	19.6 (17.4–21.9)	8	3.3 (1.4–6.4)
Nevada	24,065	21,947	91.2 (90.8–91.6)	13,255	60.4 (59.7–61.0)	6,155	46.4 (45.6–47.3)	319	5.2 (4.6–5.8)
New Hampshire	6,514	6,200	95.2 (94.6–95.7)	4,535	73.1 (72.0–74.2)	1,186	26.2 (24.9–27.5)	58	4.9 (3.7–6.3)
New Jersey	32,381	29,000	89.6 (89.2–89.9)	18,839	65.0 (64.4–65.5)	6,155	32.7 (32.0–33.3)	345	5.6 (5.0–6.2)
New Mexico	12,148	10,048	82.7 (82.0–83.4)	7,333	73.0 (72.1–73.8)	2,266	30.9 (29.8–32.0)	88	3.9 (3.1–4.8)
New York	98,746	77,673	78.7 (78.4–78.9)	45,935	59.1 (58.8–59.5)	17,587	38.3 (37.8–38.7)	975	5.5 (5.2–5.9)
North Carolina	19,970	16,307	81.7 (81.1–82.2)	11,615	71.2 (70.5–71.9)	3,923	33.8 (32.9–34.6)	249	6.3 (5.6–7.2)
North Dakota	125	109	87.2 (80.0–92.5)	79	72.5 (63.1–80.6)	—	—	—	—
Ohio	40,627	34,270	84.4 (84.0–84.7)	26,805	78.2 (77.8–78.7)	2,898	10.8 (10.4–11.2)	146	5.0 (4.3–5.9)
Oklahoma	2,240	2,057	91.8 (90.6–92.9)	1,693	82.3 (80.6–83.9)	410	24.2 (22.2–26.3)	9	2.2 (1.0–4.1)
Oregon	16,539	15,382	93.0 (92.6–93.4)	11,191	72.8 (72.0–73.5)	3,026	27.0 (26.2–27.9)	107	3.5 (2.9–4.3)
Pennsylvania	74,438	68,352	91.8 (91.6–92.0)	50,838	74.4 (74.0–74.7)	17,314	34.1 (33.6–34.5)	967	5.6 (5.2–5.9)
Rhode Island	1,849	1,593	86.2 (84.5–87.7)	1,274	80.0 (77.9–81.9)	287	22.5 (20.3–24.9)	11	3.8 (1.9–6.8)
South Carolina	13,131	12,130	92.4 (91.9–92.8)	10,176	83.9 (83.2–84.5)	2,880	28.3 (27.4–29.2)	106	3.7 (3.0–4.4)
South Dakota	882	872	98.9 (97.9–99.5)	838	96.1 (94.6–97.3)	240	28.6 (25.6–31.8)	13	5.4 (2.9–9.1)
Tennessee	36,558	32,308	88.4 (88.0–88.7)	22,882	70.8 (70.3–71.3)	5,771	25.2 (24.7–25.8)	346	6.0 (5.4–6.6)
Texas	124,728	114,732	92.0 (91.8–92.1)	77,124	67.2 (66.9–67.5)	27,982	36.3 (35.9–36.6)	1,486	5.3 (5.1–5.6)
Utah	3,853	3,126	81.1 (79.9–82.4)	2,197	70.3 (68.6–71.9)	490	22.3 (20.6–24.1)	13	2.7 (1.4–4.5)
Vermont	848	788	92.9 (91.0–94.6)	533	67.6 (64.2–70.9)	130	24.4 (20.8–28.3)	—	—
Virginia	13,852	11,639	84.0 (83.4–84.6)	8,022	68.9 (68.1–69.8)	2,706	33.7 (32.7–34.8)	125	4.6 (3.9–5.5)
Washington	24,289	22,710	93.5 (93.2–93.8)	16,823	74.1 (73.5–74.6)	7,015	41.7 (41.0–42.4)	228	3.3 (2.8–3.7)
West Virginia	11,394	10,375	91.1 (90.5–91.6)	8,241	79.4 (78.6–80.2)	797	9.7 (9.0–10.3)	43	5.4 (3.9–7.2)
Wisconsin	5,591	5,233	93.6 (92.9–94.2)	4,314	82.4 (81.4–83.5)	1,130	26.2 (24.9–27.5)	31	2.7 (1.9–3.9)
Wyoming	298	281	94.3 (91.0–96.6)	198	70.5 (64.8–75.7)	39	19.7 (14.4–25.9)	—	—

**Abbreviations:** DC = District of Columbia; HCV = hepatitis C virus.

* All 50 states and DC.

^†^ The ever-infected category was assessed during the baseline period January 1, 2013–December 31, 2021.

^§^ The viral testing, initial infection, cured or cleared, and persistent infection or reinfection categories were assessed during the follow-up period January 1, 2013–December 31, 2022.

^¶^ Using CDC guidance (https://pubmed.ncbi.nlm.nih.gov/32119076/), state-specific HCV clearance cascades were generated using the following definitions: 1) ever infected, defined as having received any positive HCV test result (reactive anti-HCV, detectable HCV RNA, or HCV genotype) during January 1, 2013–December 31, 2021 (index period); 2) viral testing, defined as having HCV RNA testing performed during January 1, 2013–December 31, 2022 (the follow-up period) for a person categorized as ever infected; 3) initial infection, defined as having received a detectable HCV RNA result during the follow-up period for any person who received viral testing; 4) cured or cleared, defined as having received a subsequent undetectable HCV RNA result during the follow-up period for any person with initial infection; and 5) persistent infection or reinfection, defined as receiving a subsequent detectable HCV RNA result during the follow-up period by person categorized as cured or cleared.

** Conditional proportion based on immediately preceding cascade step.

^††^ Dashes indicate that estimates were suppressed per National Center for Health Statistics sample guidelines. https://www.cdc.gov/nchs/data/series/sr_02/sr02-200.pdf

**FIGURE 2:**
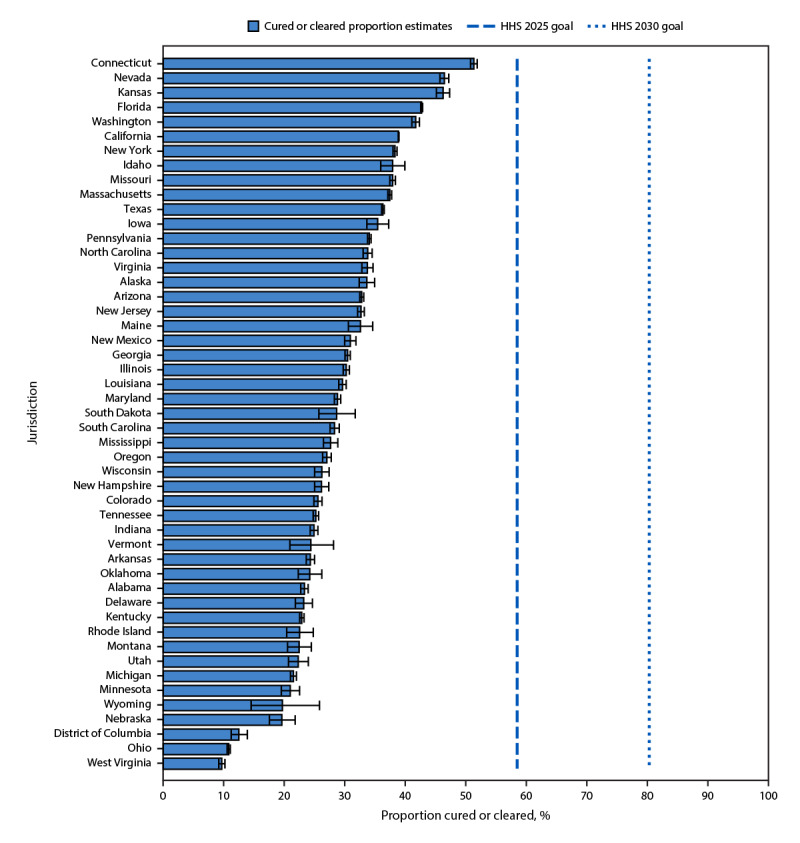
Percentage* of hepatitis C virus–infected persons with evidence of viral clearance,^†^ by jurisdiction^§^ — United States, 2013–2022^¶^ **Abbreviations:** HCV = hepatitis C virus; HHS = U.S. Department of Health and Human Services. * With 95% CIs indicated by error bars. ^†^ Based on initial infection, which was defined as having received a detectable HCV RNA result during the follow-up period for any person who received viral testing, including all persons with initial infection during January 1, 2013–December 31, 2022. ^§^ All 50 states and District of Columbia, with the exception of Hawaii and North Dakota, which are not included because the cured or cleared percentages were suppressed per National Center for Health Statistics sample guidelines. https://www.cdc.gov/nchs/data/series/sr_02/sr02-200.pdf ^¶^ The HHS 2021–2025 national strategic plan’s hepatitis C viral clearance goal is 58% by 2025 and 80% by 2030. https://www.hhs.gov/sites/default/files/Viral-Hepatitis-National-Strategic-Plan-2021-2025.pdf

By state, among those ever infected, the percentages of those who received viral testing ranged from 51% (Hawaii) to 99% (South Dakota). Among persons who received viral testing, the percentage of those who had a diagnosis of initial infection ranged from 59% (New York) to 96% (South Dakota). Among those with initial infection, the percentage of those cured or cleared ranged from 10% (West Virginia) to 51% (Connecticut). The percentage cured or cleared in 37 states was less than the estimated national average of 35%; five of the seven states with the lowest cured or cleared proportions were in southern Appalachia (West Virginia) or the north or central United States (Michigan, Minnesota, Nebraska, and Ohio). Across all jurisdictions, the percentages of HCV infections cured or cleared were below the HHS 2025 goal of 58% and well below the HHS 2030 goal of 80% (Figure 2). Finally, among those who were cured or cleared, the percentage with persistent infection or reinfection ranged from 2% (Oklahoma and Maine) to 11% (California).

## Discussion

This is the first state-specific HCV clearance cascade report, comprising data from all 50 U.S. states and DC, including approximately 1.7 million persons with evidence of a positive hepatitis C test result from a large commercial laboratory during 2013–2021, and followed through the 10-year period 2013–2022. This analysis provides insight into state-specific successes and gaps along each step of the HCV clearance cascade during the DAA treatment era.

The number of persons identified as ever having been infected with HCV varied widely by state, from 125 in North Dakota, to 338,715 in California. Multiple factors can affect these numbers, including differences in the state’s population size, the scope of laboratory coverage within the state, and hepatitis C prevalence by state.

HCV testing is necessary to distinguish past from current infection. Among persons in this cohort identified as ever having been infected (i.e., received any positive HCV test result), the median viral testing rate (having an HCV RNA test performed) was 91% across jurisdictions, reflective of recommended best practices promoting automatic HCV RNA testing for all specimens with reactive HCV antibody results.

Among states, the median percentages of persons cured or cleared (i.e., having an undetectable HCV RNA test result) was 29% (range = 10%–51%), well below both the HHS hepatitis C viral clearance goals for 2025 (at least 58%) and 2030 (at least 80%). These findings are consistent with recent studies highlighting low DAA treatment and viral clearance rates among persons with diagnosed hepatitis C infection (*9*,*10*). The proportion of cured or cleared persons also varied substantially by state (from 10% to 51%). Southern Appalachian states and most north central states had HCV cure or clearance rates below the national average, highlighting the importance of improving linkage to care and treatment coverage in these regions, which are experiencing high rates of acute hepatitis C cases in association with injection drug use (*2*).

The median state-level estimate for persistent infection or reinfection (e.g., a detectable HCV RNA test result after a previously undetectable HCV RNA test result) was 5%, ranging from 2% to 11%. Because this clearance cascade does not distinguish between persistent infection and reinfection, factors contributing to these ranges might include those affecting viral clearance (e.g., duration of infection and treatment adherence) or risk for reinfection (e.g., access to syringe services programs for persons who inject drugs), highlighting the need to investigate reasons for persistent infection and reinfection.

Development of hepatitis C viral clearance cascades is important for monitoring and identifying gaps in hepatitis C elimination efforts. Ideally, each state would have comprehensive public health hepatitis C surveillance registries, including detectable and undetectable HCV RNA results, and generate their own HCV clearance cascades. Such cascades would include results from all laboratories in a state, account for persons who moved out of state or died, and use person-level data to link individual persons to treatment and prevention services.

### Limitations

The findings in this report are subject to at least four limitations. First, the results were based on a population of persons who received a positive test result for HCV and do not represent all persons with HCV infection. Second, data from a single laboratory are not necessarily representative of a jurisdiction and characteristics of persons tested might differ by jurisdiction. Third, results for persons who received HCV laboratory testing from laboratories other than Quest Diagnostics are not represented in these estimates; inclusion of these data could lead to different estimates reported for each step. Finally, the cascade does not capture data from persons who did not receive an HCV RNA test after initial infection or after cure or clearance, which might result in underestimation of the number and proportion of persons with viral clearance or persistent viremia, respectively.

### Implications for Public Health Practice

The state-specific clearance cascades presented here facilitate the availability of data for all states, irrespective of current hepatitis C surveillance capacity to enable jurisdictional-level monitoring of hepatitis C elimination. These data demonstrate that all states have HCV clearance rates well below established national elimination goals, a finding that could serve to stimulate state-level public health action to implement best practices for diagnosing, treating, and preventing HCV infection. These practices include focusing efforts on increasing hepatitis C testing in all settings in which persons with hepatitis C receive care, ensuring unrestricted access to treatment irrespective of insurance coverage, and providing comprehensive harm reduction services for persons who use and inject drugs.

## Footnotes

*
https://www.cdc.gov/nchs/data/series/sr_02/sr02-200.pdf

† 45 C.F.R. part 46, 21 C.F.R. part 56; 42 U.S.C. Sect. 241(d); 5 U.S.C. Sect. 552a; 44 U.S.C. Sect. 3501 et seq.

## References

[1] Lewis KC, Barker LK, Jiles RB, Gupta N. Estimated prevalence and awareness of hepatitis C virus infection among U.S. adults: National Health and Nutrition Examination Survey, January 2017–March 2020. Clin Infect Dis 2023;77:1413–5. 10.1093/cid/ciad411 37417196 PMC11000503

[2] CDC. Viral hepatitis: 2022 viral hepatitis surveillance report. Atlanta, GA: US Department of Health and Human Services, CDC; 2024. https://www.cdc.gov/hepatitis/statistics/2022surveillance/index.htm

[3] Owens DK, Davidson KW, Krist AH, ; US Preventive Services Task Force. Screening for hepatitis C virus infection in adolescents and adults: US Preventive Services Task Force recommendation statement. JAMA 2020;323:970–5. 10.1001/jama.2020.1123 32119076

[4] American Association for the Study of Liver Diseases; Infectious Disease Society of America. Recommendations for testing, managing, and treating hepatitis C. Accessed May 05, 2022. Arlington, VA: American Association for the Study of Liver Diseases; the Infectious Disease Society of America; 2022. https://www.hcvguidelines.org 10.1002/hep.31060 PMC9710295 31816111

[5] Falade-Nwulia O, Suarez-Cuervo C, Nelson DR, Fried MW, Segal JB, Sulkowski MS. Oral direct-acting agent therapy for hepatitis C virus infection: a systematic review. Ann Intern Med 2017;166:637–48. 10.7326/M16-2575 28319996 PMC5486987

[6] US Department of Health and Human Services. Viral Hepatitis National Strategic Plan for the United States: a roadmap to elimination for the United States, 2021–2025. Washington, DC: US Department of Health and Human Services; 2020. https://www.hhs.gov/sites/default/files/Viral-Hepatitis-National-Strategic-Plan-2021-2025.pdf

[7] Montgomery MP, Sizemore L, Wingate H, Development of a standardized, laboratory result-based hepatitis C virus clearance cascade for public health jurisdictions. Public Health Rep 2024;139:149–53. 10.1177/00333549231170044 37140162 PMC10851908

[8] Wester C, Osinubi A, Kaufman HW, Hepatitis C virus clearance cascade—United States, 2013–2022. MMWR Morb Mortal Wkly Rep 2023;72:716–20. 10.15585/mmwr.mm7226a3 37384551 PMC10328490

[9] Thompson WW, Symum H, Sandul A, Vital signs: hepatitis C treatment among insured adults—United States, 2019–2020. MMWR Morb Mortal Wkly Rep 2022;71:1011–7. 10.15585/mmwr.mm7132e1 35951484 PMC9400534

[10] Ferrante ND, Newcomb CW, Forde KA, The hepatitis C care cascade during the direct-acting antiviral era in a United States commercially insured population. Open Forum Infect Dis 2022;9:ofac445. 10.1093/ofid/ofac445 36092829 PMC9454032

